# Book Notices

**Published:** 1899-12

**Authors:** 


### BOOK NOTICES.

A Text-Book on Practical Obstetrics. By Egbert H. Grandin, M. D., Gynæcologist to the Columbus Hospital. With the collaboration of George W. Jarman, M. D., Gynæcologist to the Cancer Hospital. Second edition. Revised and enlarged. Illustrated with sixty-four full-page photographic plates and eighty-six illustrations in the text. 6½ x 9½ inches. Pages xiv--461. Extra cloth, $4.00 net; sheep, $4.75 net. The F. A. Davis Co., Publishers, 1914-16 Cherry Street, Philadelphia.

These two authors published a book in 1894, entitled *Obstetric Surgery*, reviewed in this journal March, 1895, page 146. They issued a supplementary volume entitled *Pregnancy, Labor, and the Puerperal State* in 1895, reviewed in The Homœopathic Physician for February, 1896, page 108. The two volumes were later bound in one. The volume now before us is practically a second edition of these two combined works, and is a most excellent text-book of the subject of midwifery. Nothing more need be said, than that its plan has had imitators.

Midwifery has made considerable advances since the time of Cazeaux's great work, and while that author will never be discarded, yet we need more modern text-books to keep us aware of the progress in the art. The book under review fulfills this condition.

New, Old, and Forgotten Remedies. Papers by Many Writers. Collected, arranged, and edited by E. P. Anshutz. Philadelphia: Bœricke & Tafel, 1900. Price, cloth, $2.00; by mail, $2.20.

The author of this book is the manager of the publishing department of Bœricke & Tafel's well-known homœopathic pharmacy. He is not strictly a medical man. That is, he does not have a degree and sport a "sheep-skin." But he ought to be in the ranks of the profession which would undoubtedly be adorned and benefited by his presence. He is much more worthy of the title of "Doctor" than many a man in the profession. His pithy and incisive articles in his little journal *The Homœopathic Envoy* show well his ability. Now we have this book which is valuable for the amount of information it gives upon remedies not generally met with in the materia medica. Here they are found arranged in alphabetical order with suitable indexes, rendering them easy of reference. We cordially commend this book to the profession.

A Repertory to the Cyclopædia of Drug Pathogenesy. Compiled by Richard Hughes, M. D. Parts I, II, and III. London: E. Gould & Son, 59 Moorgate Street, E. C.; New York: Bœricke & Tafel, 159 Grand Street, 1897.

A review of this work was published in The Homœopathic Physician for September, 1897, giving its principal points. The present notice is intended more especially to jog the memories of the profession and induce them to patronize what we regard as a great work. When any member of the profession expends time and money to render more accessible the symptomatology of the materia medica, such a man should have his efforts rewarded and his hands upheld by the liberal purchase of his work by those who do nothing in the line of this kind of labor. There is not enough appreciation of this kind of work by the profession, whether by Dr. Hughes or by others, and we once more urge upon the profession the merit of the book and their need of it. All who wish to find further information of its character should refer to the review in this journal for September, 1897.

The Surgical Diseases of the Genito-Urinary Tract, Venereal and Sexual Diseases. A Text-Book for Students and Practitioners. By G. Frank Lydston, M. D., Professor of the Surgical Diseases of the Genito-Urinary Organs and Syphiology in the Medical Department of the State University of Illinois; Professor of Criminal Anthropology in the Kent College of Law; Surgeon-in-Chief of the Genito-Urinary Department of the West-Side Dispensary; Fellow of the Chicago Academy of Medicine; Fellow of the American Academy of Political and Social Science; Delegate from the United States to the International Congress for the Prevention of Syphilis and the Venereal Diseases, held at Brussels, Belgium, September 5th, 1899, etc. Illustrated with 233 engravings, 6½ x 9¾ inches; pages xvi —1024. Extra cloth, $5.00, net; sheep or half-Russia, $5.75, net. The F. A. Davis Co., Publishers, 1914-16 Cherry Street, Philadelphia.

This book is a most interesting addition to the literature of genito-urinary diseases. It is in fact a reproduction of the author's lectures on this branch of medicine in the Medical Department of the University of Illinois. There are some original ideas, which the author modestly calls heresies, taught in its pages. These heresies we do not feel competent to discuss, especially in the limited time at our command.

There are over one hundred and eighty pages devoted to syphilis alone. The theory of contagion of this terrible scourge accepted by the author is that of Dr. Fessenden N. Otis, his friend, and to whom he was hospital assistant and to whom the book is dedicated. The idea that "the contagium consists of a degraded infectious cell of very minute proportions, a view in no wise inconsistent with the germ theory, this cell having been primarily infected by the germ and acting as a carrier of infection thereafter."

The author then goes on to describe the mechanism of the infection. This description we have not space to reproduce here, though it is not only novel and ingenious, but interesting.

The author's views on sexual incontinence and depravity are also interesting but not suitable for reproduction here.

The general plan of the book is a division into ten parts. The first part being the general principles of genito-urinary pathology and therapeutics. The second parts deals with non-venereal diseases of the penis. The third part treats of the diseases of the urethra. Part four treats of chancroid and bubo, and part fifth of syphilis. The remaining parts treat of sexual physiology, of diseases of prostate, of the urinary bladder, of surgical affections of the kidneys and the testis and spermatic cord.

Keynotes and Characteristics, with Comparisons of Some of the Leading Remedies of the Materia Medica. By H. C. Allen, M. D., Professor of Materia

Medica and *The Organon* in The Hering Medical College and Hospital, of Chicago. Second edition, revised and enlarged. Philadelphia and Chicago: Bœricke & Tafel, 1899. Price, cloth, $2.00, net; by mail, $2.10.

The first edition of this book was noticed in THE HOMŒOPATHIC PHYSICIAN for January, 1899, page 40. In one short year we are presented with the second edition.

What was said by this journal before, can be said again with increased emphasis. No physician making the slighest pretension to Homœopathy should be without this book. No physician who believes in Homœopathy should be dependent upon merely casual references to its pages. He ought to commit it to memory. Its indications are needed every day in making prescriptions. These indications are the old keynotes of the late Dr. Henry N. Guernsey, invaluable in studying the simillimum. When the editor of THE HOMŒOPATHIC PHYSICIAN was a beginner in medicine thirty years ago, he *did* commit these keynotes to memory, and he has never regretted the labor for they have been of continual use ever since. Hence the admonition above, to every physician to do likewise.

Dr. Allen has put them in a most convenient form and there is no excuse for any homœopathist not to know them.

A PRACTICAL TREATISE ON THE DISORDERS OF THE SEXUAL ORGANS OF MEN. By Bukk G. Carleton, M. D., Genito-Urinary Surgeon and Specialist to the Metropolitan Hospital, etc. Second edition, revised and enlarged; 333 pages. New York: Bœricke & Runyon Co., 1900. Price, $2.50, net; by mail, $2.67.

This book shows that it is appreciated by the medical profession when a second edition is issued in two years. A review of the first edition will be found in the pages of THE HOMŒOPATHIC PHYSICIAN for February, 1899, page 92. What was said there is equally applicable now.

The therapeutics are of course objectionable for a book confessedly homœopathic. They are too strongly of the character of the old school of medicine.

The indications for the homœopathic remedies are given in the last fifty pages. They are good as far as they go, but might be much extended. As in the first edition there is no repertory to these indications. The addition of a repertory to any homœopathic book is as essential to the value and usefulness of the book as is a good general index to books in other departments of learning.

### CHRONIC DYSPEPSIA SUCCESSFULLY TREATED WITH $H_2O_2$.

By Geo. A. Gilbert, M. D., Danbury, Conn.

The case herewith subjoined is one of interest on account of its typical character, its long standing, and its speedy recovery on the adoption of a rational treatment.

Peter H., aged 40, Hungarian, farm laborer, applied for treatment at my office on July 1, 1899. He was a strapping fellow, mostly skin and bones, of about one hundred and seventy pounds' weight, and would not have been thought ill except for the prominent dark rings under his eyes, his injected conjunctivæ, and a drawn, hunted expression on his countenance, indicative of past trouble or imminent danger. The history he gave was somewhat as follows:

Six years previously, on his voyage to this country, he suffered from an attack of acute gastritis, attended with retchings of the most violent character. Soon after landing he recovered sufficiently to attend to his work; but he says he has "never been the same man since." In all this long period he has not eaten "a good square meal," nor enjoyed what he has eaten, the burning pain in the epigastrium, after meals, becoming so great occasionally that for fear of its repetition he has gone without food for two or three days at a time. Belching of enormous quantities of gas, too, is common with him soon after eating, thus evidencing the presence of undigested food with its resultant fermentation. The patient states, that in order to get relief he has spent all his wages upon various doctors, specialists, quacks, nostrums, etc., and swears that he is worse to-day than on the day he first landed in this country.

On examination it was found that he was slightly feverish, pulse rapid, tongue flabby and heavily coated, while the teeth and entire cavity of the mouth were covered with a foul-smelling, sticky mucus. That the stomach received, in the process of starch digestion, little or no assistance from the salivary glands of the mouth was plainly apparent. In deciding on the mode of treatment it was obvious that the lack of the usual amount of gastric secretion must be met by restoring the physiological conditions upon which the secretion depends. In other words, in order to relieve the inflammatory condition of the gastric mucous membrane and restore the function of the peptic glands, antiseptics were required. The patient therefore was furnished with a flask of Ozonized water, made of one part Hydrozone to four parts of water, and directed to wash out his mouth every night and morning, thoroughly cleansing the tongue, teeth, and gums of the unhealthy mucus and any pathogenic germs it might contain. To destroy the microbic elements of fermentation in the stomach and dissolve the tenacious mucus there, a mixture of one ounce of Hydrozone with two parts of sterilized water was made, and half a tumblerful directed to be taken half an hour before meals. Having thus procured a clean surface in the stomach, the patient was advised to take immediately after meals a drachm of Glycozone, diluted in a wineglassful of water, for the purpose of enhancing cellular action and stimulating healthy granulations. Of course he was ordered to select his food with care and eat regularly.

The result of this simple procedure was magical. Although for the first two or three days there was some discomfort after eating, this soon disappeared, and at the end of a fortnight the patient reported that for the first time in six years he was enabled to eat his meals without dread of subsequent distress and eructations of gas. (In the opinion of the writer the fermentation was thus quickly subdued by the active oxidation resulting from the liberation of nascent oxygen.) The treatment was continued in this manner for another month and then gradually abandoned. On September 1st, the patient came to the office, expressed his eternal gratefulness, said that he weighed 185 pounds and believed himself to be completely cured.—*New England Medical Monthly, December, 1899.*

